# *Solanum lyratum* Extracts Induce Extrinsic and Intrinsic Pathways of Apoptosis in WEHI-3 Murine Leukemia Cells and Inhibit Allograft Tumor

**DOI:** 10.1155/2012/254960

**Published:** 2012-05-07

**Authors:** Jai-Sing Yang, Chia-Chun Wu, Chao-Lin Kuo, Yu-Hsuan Lan, Chin-Chung Yeh, Chien-Chih Yu, Jin-Cherng Lien, Yuan-Man Hsu, Wei-Wen Kuo, W. Gibson Wood, Minoru Tsuzuki, Jing-Gung Chung

**Affiliations:** ^1^Department of Pharmacology, China Medical University, Taichung 404, Taiwan; ^2^Department of Biological Science and Technology, China Medical University, Taichung 404, Taiwan; ^3^School of Chinese Pharmaceutical Sciences and Chinese Medicine Resources, China Medical University, Taichung 404, Taiwan; ^4^School of Pharmacy, China Medical University, Taichung 404, Taiwan; ^5^Department of Urology, China Medical University Hospital, Taichung 404, Taiwan; ^6^Graduate Institute of Pharmaceutical Chemistry, China Medical University, Taichung 404, Taiwan; ^7^Department of Pharmacology, School of Medicine, Geriatric Research, Education and Clinical Center, VA Medical Center, University of Minnesota, Minneapolis, MN 55455, USA; ^8^Department of Biochemistry, Nihon Pharmaceutical University, Saitama 362-0806, Japan; ^9^Tsuzuki Institute for Traditional Medicine, China Medical University, Taichung 404, Taiwan; ^10^Department of Biotechnology, Asia University, Taichung 413, Taiwan

## Abstract

We investigated the molecular mechanisms of cell cycle arrest and apoptotic death induced by *Solanum lyratum* extracts (SLE) or diosgenin in WEHI-3 murine leukemia cells *in vitro* and antitumor activity *in vivo*. Diosgenin is one of the components of SLE. Our study showed that SLE and diosgenin decreased the viable WEHI-3 cells and induced G_0_/G_1_ phase arrest and apoptosis in concentration- or time-dependent manners. Both reagents increased the levels of ROS production and decreased the mitochondrial membrane potential (ΔΨ_*m*_). SLE- and diosgenin-triggered apoptosis is mediated through modulating the extrinsic and intrinsic signaling pathways. Intriguingly, the p53 inhibitor (pifithrin-*α*), anti-Fas ligand (FasL) mAb, and specific inhibitors of caspase-8 (z-IETD-fmk), caspase-9 (z-LEHD-fmk), and caspase-3 (z-DEVD-fmk) blocked SLE- and diosgenin-reduced cell viability of WEHI-3 cells. The *in vivo* study demonstrated that SLE has marked antitumor efficacy against tumors in the WEHI-3 cell allograft model. In conclusion, SLE- and diosgenin-induced G_0_/G_1_ phase arrest and triggered extrinsic and intrinsic apoptotic pathways via p53 activation in WEHI-3 cells. SLE also exhibited antitumor activity *in vivo*. Our findings showed that SLE may be potentially efficacious in the treatment of leukemia in the future.

## 1. Introduction

In Taiwan, 4.32 per 100,000 people die each year of leukemia according to the Department of Health, Executive Yuan, Taiwan in 2010s [1]. Hematopoietic stem cell transplantation, radiotherapy, and chemotherapy agents are usually used in the treatment for leukemia patients, but these outcomes are not fully satisfactory [2]. The most effective strategy for killing cancer cells is to induce cell cycle arrest and apoptosis which plays an important role as an antitumor mechanism in human leukemia cells [3]. Characteristics of apoptosis include chromatin condensation, DNA fragmentation and membrane blebbing, apoptotic bodies, translocation of phosphatidylserine (PS) of the plasma membrane [3–5], caspase cascade of cell death signaling on extrinsic, and intrinsic-dependent events that regulate proapoptotic and antiapoptotic proteins [3, 6]. Arresting tumor cells to G_0_/G_1_ phase and apoptosis may offer therapeutic possibilities for treating malignant tumors [7, 8]. It is fully reported that reagents which promoted cell cycle arrest or apoptotic induction have been used with medicinal plants [9, 10]. Numerous studies had been showed that increased consumption of plant-based diet can reduce the risk of cancer [11].

*Solanum lyratum* Thunberg (Solanaceae), one of the traditional Chinese medicines (TCM) in Taiwan and China is used to regulate immune function [12] and treat allergic responses for generations [13]. Our previous study demonstrated that *Solanum lyratum* treatment promoted the activity of phagocytosis by macrophages in the peripheral blood mononuclear cells (PBMC) and peritoneal cells from normal and leukemia mice *in vivo *[14]. We also showed that natural killer (NK) cells from the normal and leukemia mice after SLE treatment can kill the YAC-1 target cells [14]. Therefore, the regulations of immune function might be one of the antitumor mechanisms of SLE in leukemia cells. However, *Solanum lyratum *is commonly used for treatment of liver, lung esophagus cancer, and leukemia in the Chinese population [15, 16]. Many studies showed that *Solanum lyratum* extracts (SLE) inhibited proliferation in human hepatoma BEL-7402 cells, gastric carcinoma SGC-7901 cells, and melanoma A375-S2 cells *in vitro* and* in vivo *[16, 17]. SLE also triggered apoptosis in human cervical cancer HeLa cells through Fas/Fas ligand (FasL) expression [18] promoted the activity of protein kinase A (PKA) in the gastric cancer cells [19, 20] and provoked caspase-3-mediated apoptosis in human colon cancer colo 205 cells [21]. Additionally, SLE exhibited antitumor activity through caspase-8 and caspase-9 activations and mitogen-activated protein kinase (MAPK) regulation in Lewis lung carcinoma (LLC) cells [22]. The goal of this study hypothesized that whether the antileukemia activity of SLE mediates *via* its direct cytotoxic effect and explores the molecular mechanisms in WEHI-3 murine leukemia cells. Hence, the study focused on the cell cycle arrest and apoptosis-induced by SLE in the WEHI-3 cells. Based on *in vitro* and *in vivo* studies, we found that SLE inhibited cells viability induced cell apoptosis, simultaneously arresting the WEHI-3 cells to G_0_/G_1  _phase through regulating activation of p53/Fas signaling and suppressed allograft tumor *in vivo*.

## 2. Materials and Methods

### 2.1. Plant Material and Preparation of *Solanum lyratum* Extracts (SLE)

SLE was obtained from Dr. Chao-Lin Kuo (School of Chinese Pharmaceutical Sciences and Chinese Medicine Resources, China Medical University) as described previously [21]. *Solanum lyratum* was collected in September 2002 from Dongpu, Sinyi Township, Nantou County, Taiwan. The voucher specimens (CMU SL 0222) were deposited in China Medical University. The 600 g of *Solanum lyratum* was extracted frequently with 50% ethanol at room temperature. The combined all ethanol extracts were filtered and evaporated under reduced pressure then get 58.44 g of brownish viscous residue. For this experiment, the crude extracts were dissolved in dimethyl sulfoxide (DMSO) [21].

### 2.2. Chemicals and Reagents

Diosgenin, agarose, 4,6-diamidino-2-phenylindole dihydrochloride (DAPI), dimethyl sulfoxide (DMSO), propidium iodide (PI), Triton X-100, pifithrin-*α* (PFT*α*; p53 inhibitor), Tris-HCl, and ribonuclease A were obtained from Sigma-Aldrich Corp. (St. Louis, MO, USA). 2′,7′-dichlorodihydrofluorescein diacetate (H_2_DCF-DA), 3,3′-Dihexyloxacarbocyanine iodide (DiOC_6_), RPMI-1640 medium, L-glutamine, fetal bovine serum (FBS), Trypsin-EDTA, and penicillin/streptomycin were purchased from Invitrogen/Life Technologies (Carlsbad, CA, USA). Caspase-3, -8, and -9 activity assay kits, caspase-3-specific inhibitor (z-Asp-Met-Gln-Asp-fluoromethyl ketone; z-DEVD-fmk), caspase-8-specific inhibitor (z-Leu-Glu-His-Asp-fluoromethyl ketone; z-IETD-fmk), and caspase-9-specific inhibitor (z-Ile-Glu-Thr-Asp-fluoromethyl ketone; z-LEHD-fmk) were bought from R&D Systems (Minneapolis, MN, USA). Tdt-mediated deoxyuridine triphosphate nick end labeling (TUNEL) assay kit was purchased from Roche Diagnostics (GmBH, Mannheim, Germany). Annexin V/PI staining kit was bought from Serotec (Raleigh, NC, USA). These primary antibodies (anticaspase-3-FITC, anti-FasL, anti-p53, anticyclin D, anti-CDK4, anti-CDK6, anti-Fas, anti-FADD, anticytochrome c, anti-Apaf-1, anti-Bcl-2, anti-Bcl-xl, anti-Bax, anti-BAD, and anti-GAPDH), and second antibodies for Western blotting were obtained from Santa Cruz Biotechnology, Inc. (Santa Cruz, CA, USA). The primary antibodies (anticaspase-8, anticaspase-9, and anticaspase-3) were obtained from Cell Signaling Technology (Danvers, MA, USA). FITC-conjugated anti-FasL and its FITC-conjugated isotype mAb were obtained from BD Biosciences Pharmingen (San Diego, CA, USA).

### 2.3. Determination of Diosgenin from SLE by HPLC

Diosgenin is isolated from SLE as described previously [15, 23]. Standard stock solution containing 5 mg/mL of diosgenin was prepared by dissolving approximately 3.1 mg of compound in 0.62 mL methanol. HPLC analysis was performed on SHIMADZU (Japan) two solvent delivery system model CBM-20A together with a model RID-10A refractive index detector. Data acquisition was performed using SHIMADZU Class-VP software. Chromatography was carried out on a Cosmosil 5C-18 MSII column (250 × 4.6 mm i.d.). Isocratic elution was performed with water and HPLC-grade methanol (10/90, v/v) at a flow rate of 1 mL/min. The solvents were filtered through a 0.45 *μ*m of filter prior to use.

### 2.4. Cell Culture and SLE and Diosgenin Treatment

The murine myelomonocytic leukemia cell line (WEHI-3) was obtained from the Food Industry Research and Development Institute (Hsinchu, Taiwan). Cells were grown in 75-cm^2^ tissue culture flasks at 37°C under a humidified 5% CO_2_ atmosphere in RPMI 1640 medium containing 10% FBS, 2 mM L-glutamine, 100 Units/mL penicillin, and 100 *μ*g/mL streptomycin. Exponentially growing cells at 2 × 10^5^/mL were exposed to different doses of SLE or for different time points. The 0.1% DMSO is as a vehicle control. Cell morphological examination was determined utilizing a phase-contrast microscope [21].

### 2.5. Assessment for Cell Viability

Cell viability was determined by a PI exclusion method and flow cytometry [24, 25]. WEHI-3 cells (2 × 10^5^ cells/mL) in 24-well plates were incubated with 0, 100, 200, and 400 *μ*g/mL of SLE or 0, 25, 50, and 100 *μ*M of diosgenin for 24, 48 and 72 h. For incubation with the inhibitors, cells were pretreated with 10 *μ*M of caspase-3 inhibitor, caspase-9 inhibitor, and caspase-8 inhibitor for 1 h, followed by treatment with or without SLE (200 *μ*g/mL) or diosgenin (50 *μ*M). At the indicated time courses, cells were collected and then resuspended in PBS containing 4 *μ*g/mL of PI and then analyzed by flow cytometry (FACS Calibur, Becton Dickinson, Franklin Lakes, NJ, USA) [25, 26]. All experiments were performed in triplicate. Percentage of cell viability was calculated as a ratio of SLE- or diosgenin-treated cells.

### 2.6. Analysis for Cell Cycle Progression by Flow Cytometry

WEHI-3 cells (2 × 10^5^ cells/mL) in 24-well flask were exposed to 200 *μ*g/mL of SLE or 50 *μ*M of diosgenin for 0, 24, and 48 h. Cells were then collected, fixed in 70% ethanol overnight, washed in PBS once, and resuspended in 500 *μ*L of 192 mM Na_2_HPO_4_, 4 mM citric acid, pH 7.8 at 25°C for 30 min. The cells were stained with 0.5 mL of PBS containing 1 mg/mL RNase, 10 *μ*g/mL PI for 30 min in the dark and then analyzed by flow cytometry [27, 28].

### 2.7. Analysis of Apoptotic Cells by DAPI/TUNEL Double Staining

TUNEL staining was performed according to the manufacturer's protocols (*in situ* cell death detection kit; Roche Diagnostics). Cells (2 × 10^5^ cells/mL) in 24-well plates were treated without or with 200 *μ*g/mL of SLE or 50 *μ*M of diosgenin for 48 h. Cells were harvested and immediately incubated with working strength terminal deoxynucleotidyl transferase (Tdt) enzyme in a humidified chamber at 37°C for 1 h. The cells were immersed in stop/wash buffer and gently rinsed with PBS. FITC-labeled antidigoxigenin antibody was then applied to cells and incubated at 37°C for 30 min in the dark. Cells were washed in PBS, stained with DAPI, and mounted with DABCO (Sigma-Aldrich). DAPI- and TUNEL-positive cells were visualized with a fluorescence microscope [29, 30].

### 2.8. Assay of Early Apoptotic Cells by Annexin V/PI Double Staining

Cells (2 × 10^5^ cells/mL) in 24-well plates were incubated with or without 200 *μ*g/mL of SLE or 50 *μ*M of diosgenin for 12 h, the cells were washed twice with PBS and resuspended in binding buffer (10 mM HEPES/NaOH (pH 7.4), 140 mM NaCl and 2.5 mM CaCl_2_). The cells were then stained with fluorescein isothiocyanate (FITC)-Annexin V and PI for 30 min in the dark at room temperature according to the manufacturer's directions (Serotec Inc., Raleigh, NC, USA). The fluorescence intensity of cells was immediately analyzed by flow cytometry [21, 31, 32].

### 2.9. Detection of the Fas Ligand Expression by Flow Cytometry

Fas ligand (FasL) cell surface antigen expression was measured by flow cytometry as previously described [33, 34]. SLE at 200 *μ*g/mL or diosgenin at 50 *μ*M treated cells were rinsed in PBS. FasL was analyzed by direct immunofluorescence staining. FITC-conjugated anti-FasL and its FITC-conjugated isotype mAb (BD Biosciences Pharmingen, San Diego, CA, USA) that were from examined cells were analyzed using a flow cytometer [33, 34].

### 2.10. Treatment with Anti-Fas Ligand (FasL) mAb or Pifithrin-*α* (p53 Inhibitor)

Cells (2 × 10^5^ cells/mL) in 24-well plates were pretreated with 50 ng/mL of anti-Fas ligand (FasL) mAb or 10 *μ*M of pifithrin-*α* (p53 inhibitor) for 1 h, followed by treatment with or without 200 *μ*g/mL of SLE or 50 *μ*M of diosgenin for 48 h. FasL blocking experiments were performed by treatment with Fas ligand (FasL) mAb (BD Biosciences Pharmingen, San Diego, CA, USA; 500 ng/mL) or a mouse IgG1 isotype-matched control mAb (BD Biosciences Pharmingen; 500 ng/mL) as previously described [33, 34].

### 2.11. Flow Cytometric Detections of Reactive Oxygen Species (ROS) and Mitochondrial Membrane Potential ΔΨ_*m*_

Cells (2 × 10^5^ cells/mL) in 24-well plates were exposed to 200 *μ*g/mL of SLE or 50 *μ*M of diosgenin for 0, 6, 12, 24, and 48 h. ROS and ΔΨ_*m*_ were assessed by cell permeable probes H_2_DCF-DA (10 *μ*M) and DiOC_6 _(500 nM), respectively. Cells were washed with PBS and resuspended in PBS then analyzed at FL1 channel (530 nm) by flow cytometry [26, 28].

### 2.12. Immunofluorescence Staining

Cells (2 × 10^5^ cells/mL) in 4-well chamber slides were incubated with SLE (200 *μ*g/mL) or diosgenin (50 *μ*M) for 48 h. After SLE treatment, cells were fixed with iced methanol, blocked with 2% BSA, stained with anticaspase-3 monoclonal antibodies (Santa Cruz Biotechnology, Inc.) and then FITC-conjugated anti-mouse IgG antibody (Santa Cruz Biotechnology, Inc.). The cells were analysed with a fluorescence microscope [35, 36].

### 2.13. Caspase-8 and Caspase-9 Activities Assay

Cells (total 2.5 × 10^6^ cells) in 6-well plates were treated with 200 *μ*g/mL of SLE or 50 *μ*M of diosgenin for 0, 12, 24, 36, and 48 h. Cells were harvested and lysed in a lysis buffer (50 mM Tris-HCl (pH 7.4), 1 mM EDTA, 10 mM EGTA, 10 mM digitonin, and 2 mM DTT). Cell lysates (50 *μ*g protein) were incubated with caspase-9 and -8 specific substrates (Ac-LEHD-pNA, and Ac-IETD-pNA) (R&D Systems, Inc., Minneapolis, MN, USA) for 1 h at 37°C. The caspase activity was determined by measuring OD_405_ of the released pNA [35, 37].

### 2.14. Western Blotting Analysis

Cells (total 1 × 10^7^ cells) in 75-T flask were incubated with or without SLE (200 *μ*g/mL) or diosgenin (50 *μ*M) for 0, 12, 24, and 48 h. Total protein was prepared and determined as previously described [21, 27]. Protein lysates were sonicated and the supernatants were boiled in SDS sample buffer for 5 min. The protein concentration was measured by using a BCA assay kit (Pierce Chemical, Rockford, IL, USA). Equal amounts of cell lysate were run on 10 to 12% SDS-polyacrylamide gel electrophoresis and electrotransferred to a nitrocellulose membrane by using the iBot Dry Blotting System (Invitrogen/Life Technologies). The transferred membranes were blocked for 1 h in 5% nonfat dry milk in Tris-buffered saline/Tween 20 and incubated with primary antibodies at 4°C overnight. Membranes were washed three times with Tris-buffered saline/Tween 20 for 10 min and incubated with secondary HRP-conjugated antibody [25, 29, 38]. The blots were developed by using an ECL kit and Kodak Bio-MAX MR film (Eastman Kodak, Rochester, NY, USA).

### 2.15. WEHI-3 Murine Leukemia Cells Allograft Model and *In Vivo* Antitumor Activity Assay

Eighteen BALB/c mice (4–6 weeks of age) were obtained from the National Laboratory Animal Center (NLAC, Taipei, Taiwan). All mice were fed a commercial diet and water. WEHI-3 cells (total 1 × 10^7^ cells) were resuspended in serum-free RPMI medium 1640 with BD Matrigel basement membrane matrix (BD Biosciences) at a 1 : 1 ratio (total volume 200 *μ*L). WEHI-3 cells were subcutaneously injected into the flanks of mice. Tumor mass was measured every 4 days. When tumors reached an approximate volume of 100 mm^3^, mice were selected and distributed for drug studies (day 0). Animals with tumors were randomly assigned to three treatment groups. Animals (six mice/group) were given vehicle control (olive oil), SLE (5 and 15 mg/kg) by oral gavage for QD treatment [14]. Body weight and tumors volume were measured every 4 days with a caliper. Tumor volumes were determined by measuring the length (*l*) and the width (*w*), and the volumes were calculated as *l*/*w* ^2^/2. The mice were sacrificed when the tumor burden was less than 1800 mm^3^ (day 28) [39–42]. All experiments were conducted according to the Institutional Animal Care and Use Committee (IACUC; Affidavit of Approval of Animal Use Protocol, No. 98–129-N), China Medical University (Taichung, Taiwan).

### 2.16. Statistical Analysis

All the statistical results were expressed as the mean ± S.E.M. of triplicate samples. Statistical analyses of data were done using one-way ANOVA followed by Student's* t*-test, and *P* < 0.05 were considered significant.

## 3. Results

### 3.1. HPLC Analysis in SLE

**Figure 1:**
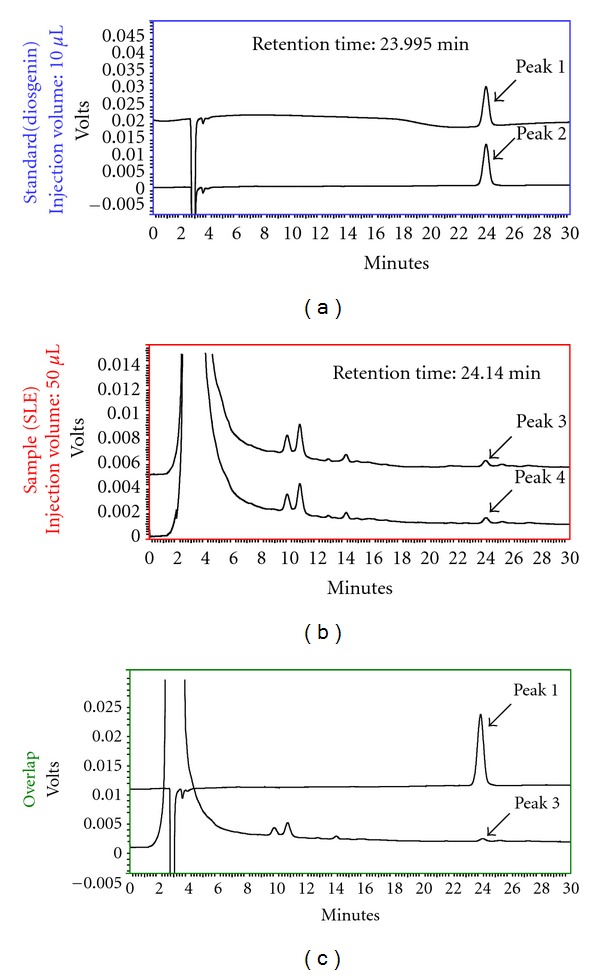
The content of diosgenin in SLE was analyzed by HPLC. HPLC was performed on SHIMADZU (Japan) two solvent delivery system model CBM-20A together with a model RID-10A refractive index detector. Data acquisition was performed using SHIMADZU Class-VP software. Chromatography was carried out on a Cosmosil 5C-18 MSII column (250 × 4.6 mm i.d.). Isocratic elution was performed with water and HPLC-grade methanol (10/90, v/v) at a flow rate of 1 mL/min. Pure diosgenin (peak 1 and peak 2) showed a retention time at 23.995 min (top), SLE (peak 3 and peak 4) showed a retention time at 24.140 min. (middle) and overlapping analysis (bottom).

The previous studies have demonstrated that diosgenin is one of the major components of the SLE [15, 23]. HPLC chromatogram of SLE analyzed using a Cosmosil 5C-18 MSII column (250 × 4.6 mm i.d.) eluted with methanol/water (90/10, v/v) at a flow rate of 1.0 mL/min and with refractive index detector. The peak at 24.140 min was identified as diosgenin as seen in Figure 1.

### 3.2. SLE and Diosgenin Inhibited Cell Proliferation, Promoted G_0_/G_1_ Phase Arrest, and Induced Cell Death in WEHI-3 Cells

**Figure 2:**
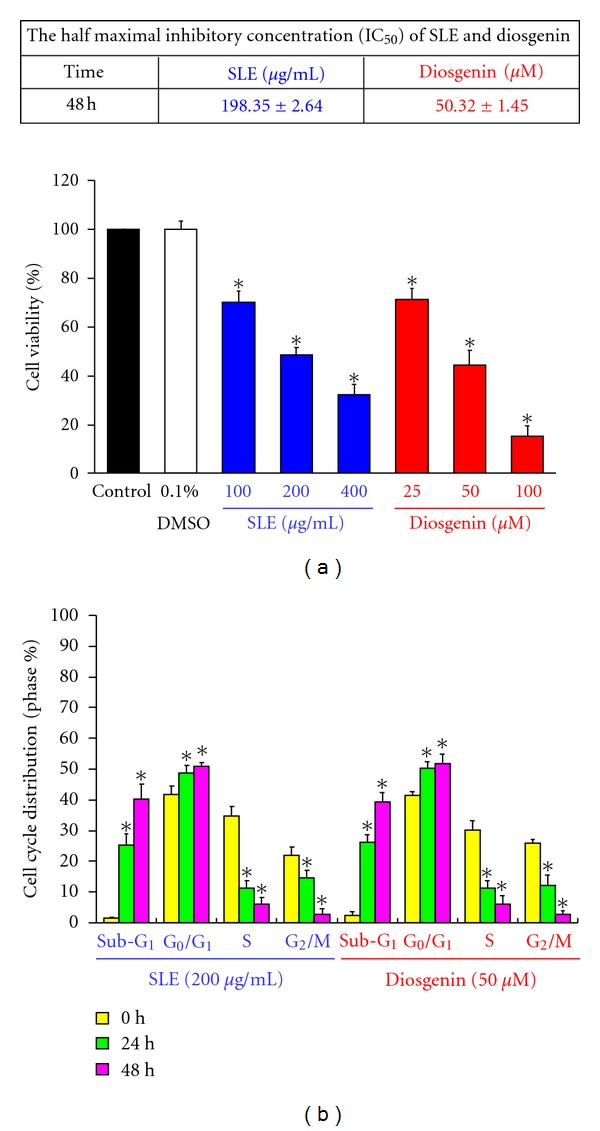
The effects of SLE and diosgenin on cell viability and cell cycle distribution in WEHI-3 cells. (a) Cells were treated with SLE (0, 100, 200, and 400 *μ*g/mL) or diosgenin (0, 25, 50, and 100 *μ*M) for 48 h. Percentage of viable cells was determined by PI exclusion method. Data are presented as the mean ± S.E.M. of three independent experiments. *, *P* < 0.05, significantly different compared with control treatment. (b) Cells were treated with 200 *μ*g/mL of SLE or 50 *μ*M of diosgenin for 24 and 48 h. The cell cycle distribution was determined using flow cytometric analysis and cell cycle distribution was quantified. Data are presented as the mean ± S.E.M. of three independent experiments. *, *P* < 0.05, significantly different compared with 0 h treatment.

We initially assessed the cell viability in WEHI-3 cells. In Figure 2(a), the concentrations of 100, 200, and 400 *μ*g/mL of SLE or 25, 50, and 100 *μ*M of diosgenin decreased the percentage of viable cells, and these effects are in a concentration-dependent manner. The half maximal (50%) inhibitory concentration (IC_50_) for a 48 h treatment of SLE and diosgenin in WEHI-3 cell was 198.35 ± 2.64 *μ*g/mL and 50.32 ± 1.45 *μ*M, respectively. Therefore, SLE at 200 *μ*g/mL or diosgenin at 50 *μ*M was selected for further experiments in this study. In addition, both reagents induced the strong growth-inhibitory and cell death effects in WEHI-3 cells. The further studies were conducted to investigate the possible mechanisms addressing cell cycle arrest or cell death by inhibitory effects in SLE- and diosgenin-treated WEHI-3 cells. Our results demonstrated that SLE and diosgenin induced G_0_/G_1_ phase arrest and cell death (sub-G1 phase) in 24 and 48 h treatments, and these effects occurred in a time-dependent manner (Figure 2(b)).

### 3.3. SLE and Diosgenin Induced Apoptotic Death and Caspase-3 Activation in WEHI-3 Cells

**Figure 3:**
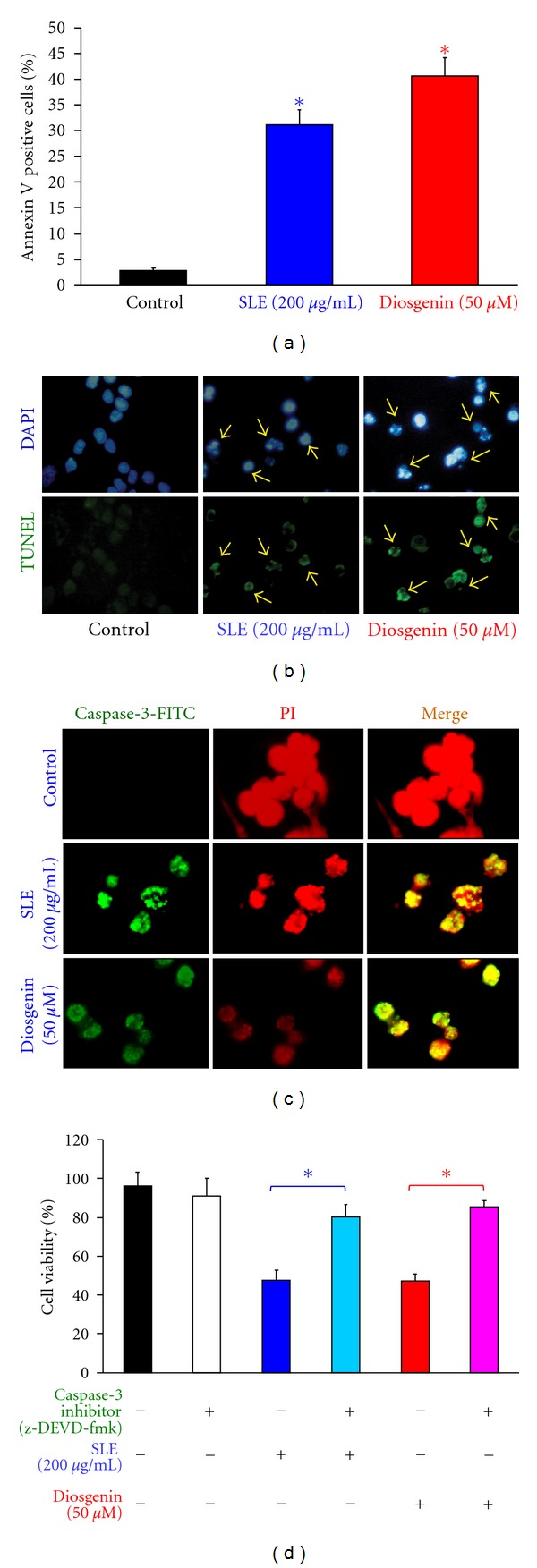
SLE- and diosgenin-induced apoptosis and caspase-3 activation in WEHI-3 cells. Cells were treated with 200 *μ*g/mL of SLE or 50 *μ*M of diosgenin for 12 h. (a) Annexin V/PI analysis was determined by flow cytometric assay. Apoptotic cell population (Annexin V positive cells) was quantified as described in materials and methods. Data are presented as the mean ± S.E.M. of three independent experiments.*, *P* < 0.05, significantly different compared with control treatment. Cells were treated with 200 *μ*g/mL of SLE or 50 *μ*M of diosgenin for 48 h. (b) DAPI/TUNEL analysis and (c) caspase-3 protein location were determined by immunostaining and photographed by fluorescence microscopic systems as described in materials and methods (400X) (↑DNA fragmentation). (d) Cells were pretreated with specific inhibitor of caspases-3 (z-DEVD-fmk) for 1 h after exposure to SLE (200 *μ*g/mL) or diosgenin (50 *μ*M) for 48 h exposure. The cells were collected to determine the percentage of viable cells. Data are presented as the mean ± S.E.M. of three independent experiments. *, *P* < 0.05, significantly different compared with SLE-treated cells.

To determine whether SLE *and diosgenin* can induce cell apoptosis, WEHI-3 cells treated with 200 *μ*g/mL of SLE or 50 *μ*M of diosgenin in WEHI-3 cells for 12 h triggered the translocation of phosphatidylserine (PS) from inner side of the plasma membrane to the outer layer of the cell membrane which was examined by Annexin V/PI analysis (Annexin positive cells: 32.89 ± 2.26% and 41.93 ± 3.50%) as shown in Figure 3(a).  Figure 3(b) showed that chromatin condensation and DNA fragmentation were evident in WEHI-3 cells treated with 200 *μ*g/mL of SLE or 50 *μ*M of diosgenin for 48 h. To investigate if SLE- and diosgenin-induced apoptosis is mediated through caspase-3-dependent pathway, WEHI-3 cells were treated with or without SLE (200 *μ*g/mL) or diosgenin (50 *μ*M) for 48 h and then stained with anticaspase-3 antibody. Our results shown in Figure 3(c) indicated that the level of caspase-3 was increased in nuclei in WEHI-3 cells after exposure to SLE or diosgenin. Cells were pretreated with caspase-3 specific inhibitor (z-DEVD-fmk) and then exposed to SLE or diosgenin. Results showed that there is a significant increase of cell viability in comparison to SLE and diosgenin (Figure 3(d)) treatment alone sample. Our findings suggest that SLE- and diosgenin-induced apoptosis was involved in caspase-3-dependent pathway in WEHI-3 cells.

### 3.4. SLE and Diosgenin Induced Apoptosis through Extrinsic Apoptotic Pathway in WEHI-3 Cells

**Figure 4:**
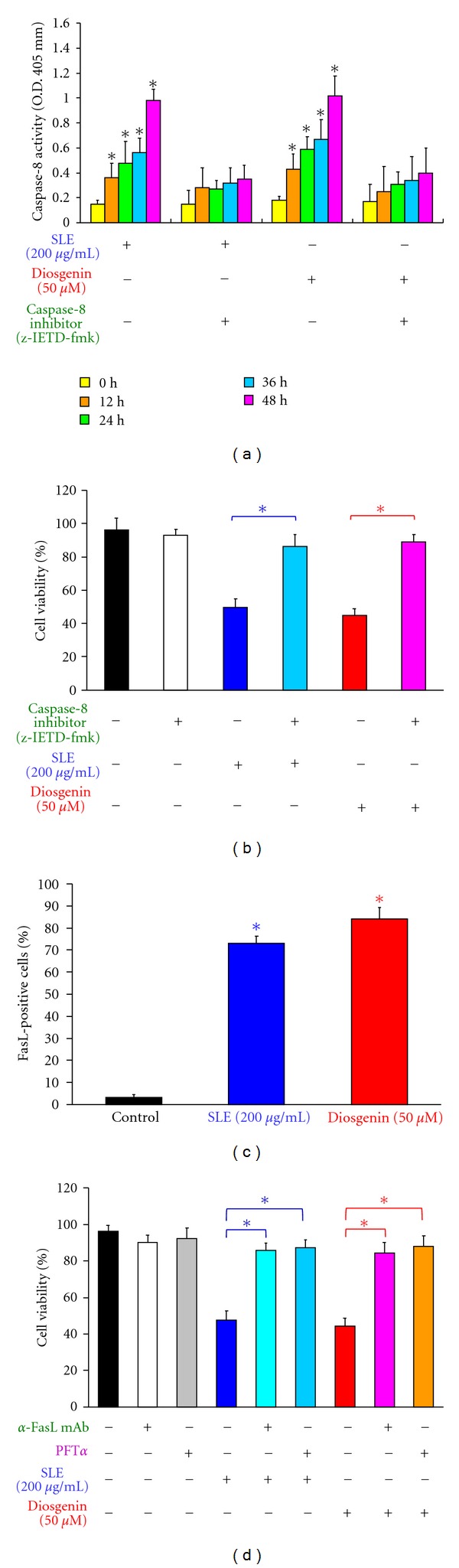
Effects of SLE and diosgenin on WEHI-3 cells in the extrinsic apoptotic pathway. Cells were pretreated with specific inhibitors of caspases-8 (z-IETD-fmk) for 1 h after exposure to SLE (200 *μ*g/mL) or diosgenin (50 *μ*M) for 12, 24, 36, and 48 h. (a) The whole-cell lysates were subjected to caspase-8 activity and (b) cells were collected after SLE or diosgenin for a 48 h treatment to determine the percentage of viable cells. (c) Cells were incubated with 200 *μ*g/mL of SLE or 50 *μ*M of diosgenin for 24 h, and FasL protein expression was detected by immunostaining and analysis by flow cytometry. (d) Cells were pretreated with anti-FasL mAb or specific inhibitor of p53 (PFT*α*) for 1 h after exposure to SLE (200 *μ*g/mL) or diosgenin (50 *μ*M) for a 48 h exposure. Cells were collected to determine the percentage of viable cells. Data are presented as the mean ± S.E.M. of three independent experiments. *, *P* < 0.05, significantly different compared with SLE-treated cells.

It is well known that caspase-3 can be activated in two major apoptotic pathways, the death-receptor (extrinsic pathway), and mitochondria-mediated (intrinsic pathway) pathways [3, 6]. Our results showed that SLE- and diosgenin-induced apoptosis might be through caspase-3-dependent signaling (Figures 3(c) and 3(d)). Thus, we determined whether the death receptor (extrinsic pathways) contributes to SLE- and diosgenin-induced apoptosis.  Figure 4(a) indicates that SLE (200 *μ*g/mL) and diosgenin (50 *μ*M) treatments time dependently caused caspase-8 activity. Cells were pretreated with caspase-8-specific inhibitor (z-IETD-fmk) and then exposed to SLE or diosgenin, the result showed that there is a significant decrease in caspase-8 activity (Figure 4(a)) and an increase in cell viability (Figure 4(b)) in comparison to SLE or diosgenin treatments alone sample.  Figure 4(c) revealed that FasL protein level was increased in SLE- and diosgenin-treated WEHI-3 cells by flow cytometry. It is reported that p53-inducible proapoptotic genes triggered apoptosis through death-receptor apoptotic pathway [43, 44]. Cells were pretreated with p53 inhibitor (pifithrin-*α*; PFT*α*) or anti-FasL mAb and then exposed to SLE or diosgenin.  Figure 4(d) showed that there is a significant increase in cell viability when in comparison to SLE- or diosgenin-treated alone samples. Our results suggest that SLE- and diosgenin-induced apoptosis might fully carried out through p53-mediated extrinsic apoptotic pathways in WEHI-3 cells.

### 3.5. SLE and Diosgenin Triggered Apoptosis through Intrinsic Pathway in WEHI-3 Cells

**Figure 5:**
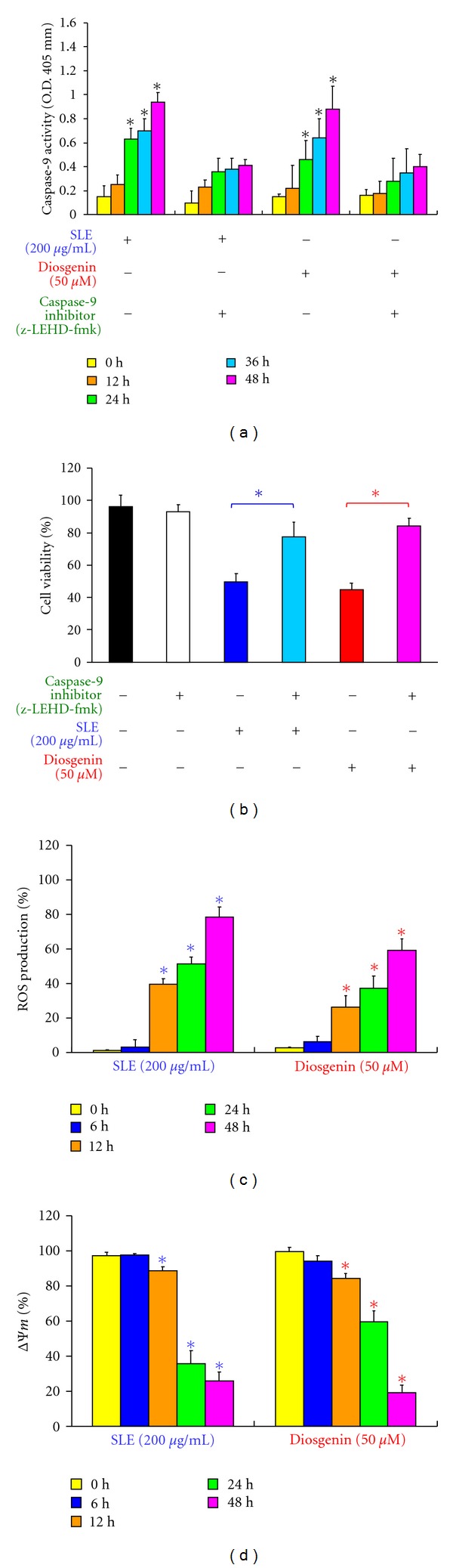
Effects of SLE and diosgenin on WEHI-3 cells in the intrinsic apoptotic pathway. Cells were pretreated with specific inhibitor of caspases-9 (z-LEHD-fmk) for 1 h after exposure to SLE (200 *μ*g/mL) or diosgenin (50 *μ*M) for 12, 24, 36, and 48 h. (a) The whole-cell lysates were subjected to caspase-9 activity assay and (b) cells were collected after SLE or diosgenin for a 48 h treatment to determine the percentage of viable cells. Data are presented as the mean ± S.E.M. of three independent experiments. *, *P* < 0.05, significantly different compared with SLE treatment. (c) The reactive oxygen species (ROS) of SLE- or diosgenin-treated WEHI-3 cells from each time point were measured by staining with H_2_DCF-DA. (d) The mitochondrial membrane potential (ΔΨ_*m*_) of both reagents-treated WEHI-3 cells was measured by staining with DiOC_6_. Data are presented as the mean ± S.E.M. of three independent experiments. *, *P* < 0.05, significantly different compared with 0 h treatment.

We determined the mitochondrial apoptotic signals if contribute to SLE- or diosgenin-induced apoptosis. In Figure 5(a), the results showed that SLE (200 *μ*g/mL) or diosgenin (50 *μ*M) stimulated caspase-9 activity in a time-dependent effect in WEHI-3 cells. Cells were pretreated with caspase-9-specific inhibitor (z-LEHD-fmk) and then exposed to SLE or diosgenin, the result showed that there is a significant decrease in caspase-9 activity (Figure 5(a)) and an increase in cell viability (Figure 5(b)) in comparison to SLE or diosgenin treatments alone cells. We examined the effects of SLE and diosgenin on the ROS production and ΔΨ_*m*_. Both reagents promoted ROS production (Figure 5(c)) and loss of ΔΨ_*m*_ (Figure 5(d)) after 12 h treatment in WEHI-3 cells. Cells were pretreated with *N*-acetylcysteine (NAC, ROS scavenger) and treated with SLE. We found that the increased the percentage of viable WEHI-3 cells occurred when compared SLE and diosgenin-treated cells (data not shown). Our findings proposed that SLE- and diosgenin-induced apoptosis was through ROS production and intrinsic pathways in WEHI-3 cells.

### 3.6. Effects of SLE and Diosgenin on G_0_/G_1_ Phase and Apoptosis-Associated Protein Levels in WEHI-3 Cells

**Figure 6:**
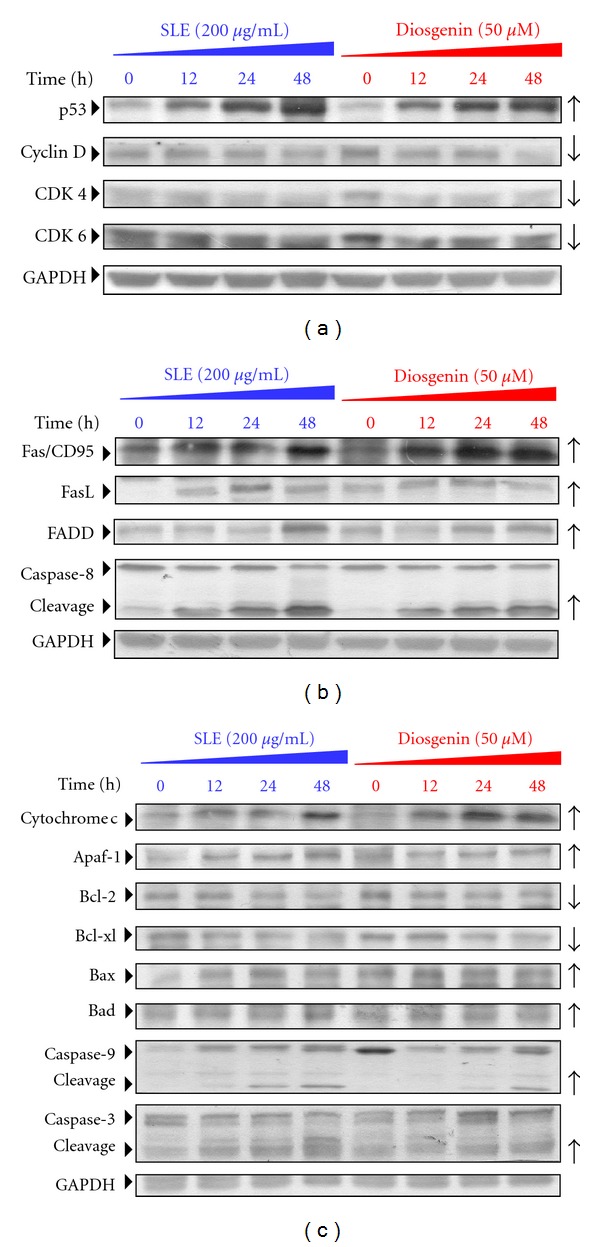
SLE and diosgenin altered the levels of G_0_/G_1_ phase and apoptotic relative proteins in WEHI-3 cells. Cells were exposed to SLE (200 *μ*g/mL) or diosgenin (50 *μ*M) and then incubated for 12, 24, and 48 h. The protein levels of (b) p53, cyclin D, CDK4, and CDK6, (b) Fas/CD95, FasL, FADD, Caspase-8, and GAPDH (b), and (c) cytochrome *c*, Apaf-1, Bcl-2, Bcl-xl, Bax, Bad, caspase-9, caspase-3, and GAPDH in SLE-treated WEHI-3 cells were determined by Western blotting. Data are presented as the mean ± S.E.M. of three independent experiments. *, *P* < 0.05, significantly different compared with 0 h treatment.

We investigated the protein levels of the G_0_/G_1_ phase and apoptosis by Western blotting. As shown in Figure 6(a), SLE and diosgenin caused an increase in the protein level of p53 and decreased the protein levels of CDK4, CDK6, and cyclin D in WEHI-3 cells. Results shown in Figure 6(b) indicated that SLE and diosgenin increased the death receptor pathway-associated protein levels, including Fas/CD95, FasL, FADD, and cleavage-caspase-8. Furthermore, mitochondrial pathway-related protein levels (cytochrome *c*, Apaf-1, Bax, Bad, and cleavage-caspase-9) were increased, but the levels of Bcl-2 and Bcl-xl were decreased in SLE and diosgenin-treated WEHI-3 cells (Figure 6(c)).

### 3.7. Antitumor Activity of SLE in WEHI-3 Cells Allograft Model

**Figure 7:**
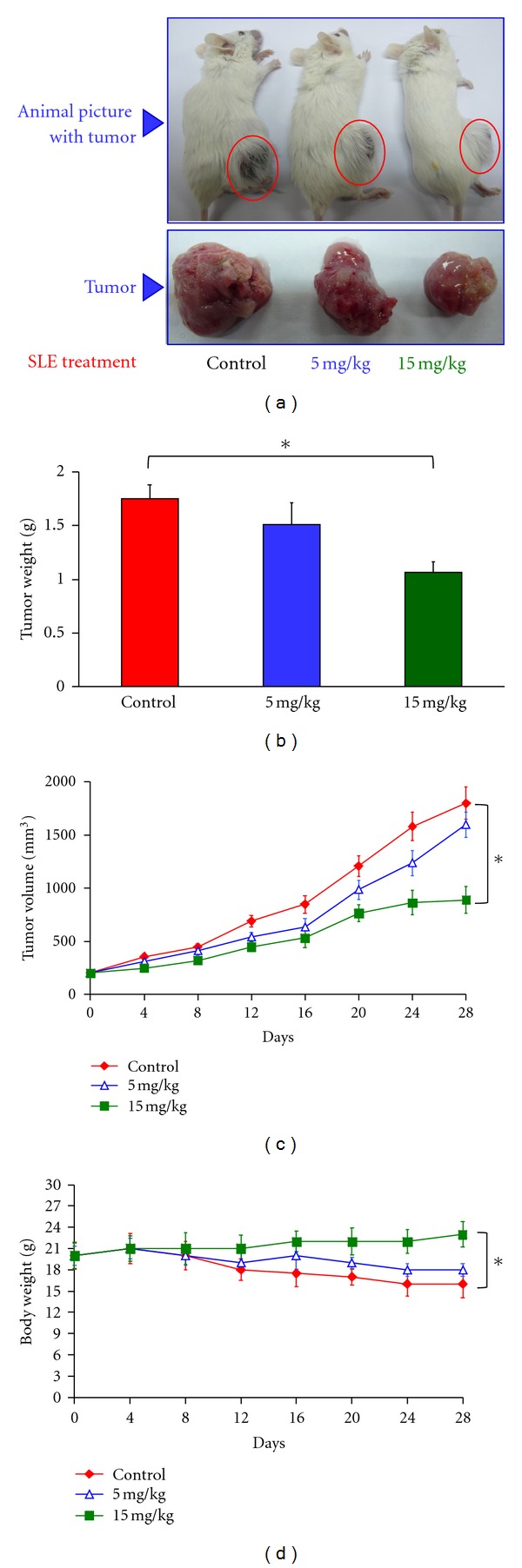
SLE inhibited tumor growth in the WEHI-3 cells allograft model. Eighteen BALB/c mice were subcutaneously implanted with 1 × 10^7^ WEHI-3 cells. When tumors reached the volume of 100 mm^3^, the mice were randomly divided into three groups (six mice/group). Group 1 was orally treated with control vehicle (olive oil) daily; group 2 was orally treated with 5 mg/kg of SLE daily; group 3 was orally treated with 15 mg/kg of SLE daily. At day 28, all animals were sacrificed. Representative (a) animals with tumor, (b) tumor weight, (c) solid tumor volume, and (d) body weight from each animal were shown. Data are presented as the mean ± S.E.M. of six animals at day 0 to 28 after tumor implantation. *, *P* < 0.05, significantly different compared with control.

Based on our *in vitro *studies, we examined the *in vivo* antitumor activities of SLE in a BALB/c mouse WEHI-3 allograft model [42]. Representative tumor weights from the WEHI-3 allograft mice treated with or without SLE were shown in Figure 7(a) and 7(b). SLE (15 mg/kg; QD; oral) significantly decreased the tumor weight by 56.84% compared with control mice. As seen in Figure 7(c), SLE (5 and 15 mg/kg; QD; oral) reduced tumor volume compared with control after treatment from day 12 to 28.  Figure 7(d) shows that the body weights of the allograft mice were not significantly different after treatment with SLE (5 and 15 mg/kg; QD; oral) from day 0 to 28. SLE significantly prevented the loss of body weight compared with the control group. Our results proposed that SLE reduced the tumor size and processed antitumor activity in the WEHI-3 allograft model.

## 4. Discussion

Traditional Chinese medicines (TCM) were appiled to prevent or therapy various tumor are marked with their high antitumor activity but low toxicity in normal cells [9, 10]. SLE is an active TCM with various immune and pharmacological effects on antitumor activity [14–16]. Our earlier study has verified that SLE affected immune response *in vivo* [14]. Herein, we further evaluated the antileukemia effects of SLE and diosgenin on WEHI-3 cells* in vitro*. Our results demonstrated that SLE significantly inhibited the viability in WEHI-3 cells (Figure 2(a)). Importantly, SLE had a relative low toxicity effect in normal PBMC cells, which suggested that SLE might be an effective and safe antitumor reagent [14]. In our study, the inhibition of tumor growth effect of SLE on the WEHI-3 cell allograft model in the SLE-treated mice was observed (Figure 7). Due to these observations, SLE is safety as a leukemia therapeutic reagent. In addition, our novel findings showed that SLE exhibited two important mechanisms in antileukemia effects; one is immune regulation [14] and the other is direct cytotoxicity effects and cell growth through induction of G_0_/G_1_ phase arrest and apoptosis in WEHI-3 cells.

It has been reported that several mechanisms for SLE-mediated inhibition of tumor cell survival and induction of apoptosis occurred in human cancer cells [16–21]. In our previous study, production of ROS in colo 205 colon cancer cells after SLE treatment might be involved in a direct anticancer effect, inhibition of cell proliferation and induction of apoptosis [21]. Herein, we characterized the effects of SLE on WEHI-3 cells. Modulation of expression and function of cell cycle regulatory proteins such as cyclin-dependent kinase inhibitor (CKI), cyclins, and CDK provides a crucial mechanism for the control of cell growth [45, 46]. In regulation of transition from G_1_ to S, activation of CDK4, CDK6, and cyclin D is one of the important mechanisms [47]. SLE induced G_0_/G_1_ phase arrest (Figure 2(b)) and inhibited the cyclin D, CDK4, and CDK6 protein levels, but it exhibited p53 protein level which was associated with cell cycle arrest (Figure 6(a)) in WEHI-3 cells. The function of p53 plays an important role in inducing cell cycle checkpoints and apoptosis in human and murine cells following ROS-induced DNA damage [48]. Our results demonstrated that induction of p53 by SLE not only caused G_0_/G_1_ phase arrest but also triggered apoptosis in WEHI-3 cells. Pretreatment with PFT*α* in SLE-treated WEHI-3 cells restored the cell viability (Figure 4(d)), suggesting the functional involvement in p53-dependent pathway. To confirm this hypothesis, further investigations such as those using p53-specific siRNA are needed.

Apoptosis is important in normal cell homeostasis and apoptosis induction is one of the best strategies for cancer treatment [4, 49]. Apoptotic signaling pathway can be divided into: (i) the death receptor includes Fas/FasL, tumor necrosis factor (TNF)/TNF receptors, and death receptor 4, 5 (DR4, 5)/DR4, 5 receptors and their downstream molecules are caspases-8 [4, 6]; (ii) the mitochondria-mediated pathway shows apoptotic stimuli induced by radiation, ROS production, and chemotherapy. Loss of ΔΨ_*m*_ could cause cytochrome *c*, Apaf-1, procaspase-9 releases and lead to the activation of the caspase cascade [4]. However, the cross-talk between these two apoptotic pathways also exists [11, 37, 50, 51]. In this study, we observed that SLE induced cell death with characteristics of apoptosis (Figure 3). Our data showed that the activities and protein levels of caspase-8, -9, and -3 were increased in SLE-treated WEHI-3 cells in a time-dependent manner (Figures 3–5). The extrinsic and intrinsic apoptotic pathways-associated proteins (Figure 6) were changed, which subsequently promoted caspase-8 and caspase-9 activation to activate downstream effectors caspase-3 in SLE-treated WEHI-3 cells. Lee et al. reported that hexane fraction of *Solanum lyratum* herba induced apoptosis through activated caspase-8, -9, and -3 proteins in LLC cells [22]. The previous studies [18–20, 22] supported our suggestions to show that SLE induced apoptosis in WEHI-3 cells through both extrinsic- and intrinsic-dependent signaling pathways.

The present study also investigated diosgenin, a food saponin and one of the compounds isolated from SLE [15, 23, 52], whether affects murine leukemia WEHI-3 cells. Importantly, we also found that there are many similar results in diosgenin-treated WEHI-3 cells when compared with that in WEHI-3 cells after SLE exposure. Based on these findings, diosgenin-provoked apoptosis in WEHI-3 cells is involved in the p53-regulated both extrinsic and intrinsic signaling pathways. This report is agreed with other studies described by Bertrand et al. regarding diosgenin treatment that triggered subsequent extrinsic and intrinsic apoptotic signaling in human erythroleukemia (HEL) cells [52, 53]. Moreover, our data shown in Figure 2(b) is consistent with the previous research that diosgenin promoted G_1_/G_0_ phase arrest in primary human thyrocytes [49, 54]. Intriguingly, Srinivasan et al. stated that diosgenin processed chemotherapeutic effects through targeting Akt-mediated NF-*κ*B and MAPK (prosurvival) signaling in human breast carcinoma (BCa) cells [49]. Hence, we suggest that SLE and diosgenin might be as potent anticancer agents for treating leukemia patients.

In summary, we demonstrated the molecular mechanisms underlying SLE-induced antitumor activity in WEHI-3 murine leukemia cells *in vitro* and* in vivo*. SLE inhibited cell proliferation in WEHI-3 murine leukemia cells through G_0_/G_1_ phase arrest, extrinsic- and intrinsic-related pathways, which is involved in p53 activation. Understanding the manner of SLE-affected cell cycle progression and triggered apoptotic pathways will facilitate the development of treatment of leukemia in the future.
